# The cellular model for Alzheimer's disease research: PC12 cells

**DOI:** 10.3389/fnmol.2022.1016559

**Published:** 2023-01-04

**Authors:** Danni Xie, Ting Deng, Zhenwei Zhai, Tao Sun, Ying Xu

**Affiliations:** ^1^State Key Laboratory of Southwestern Chinese Medicine Resources, School of Pharmacy, Chengdu University of Traditional Chinese Medicine, Chengdu, China; ^2^School of Medical Information Engineering, Chengdu University of Traditional Chinese Medicine, Chengdu, China; ^3^TCM Regulating Metabolic Diseases Key Laboratory of Sichuan Province, Hospital of Chengdu University of Traditional Chinese Medicine, Chengdu, China

**Keywords:** Alzheimer's disease, PC12 cells, culture condition, differentiation methods, transfection methods, drugs inducing AD, *in vitro* cellular models used in parallel with PC12 cells

## Abstract

Alzheimer's disease (AD) is a common age-related neurodegenerative disease characterized by progressive cognitive decline and irreversible memory impairment. Currently, several studies have failed to fully elucidate AD's cellular and molecular mechanisms. For this purpose, research on related cellular models may propose potential predictive models for the drug development of AD. Therefore, many cells characterized by neuronal properties are widely used to mimic the pathological process of AD, such as PC12, SH-SY5Y, and N2a, especially the PC12 pheochromocytoma cell line. Thus, this review covers the most systematic essay that used PC12 cells to study AD. We depict the cellular source, culture condition, differentiation methods, transfection methods, drugs inducing AD, general approaches (evaluation methods and metrics), and *in vitro* cellular models used in parallel with PC12 cells.

## Introduction

Alzheimer's disease (AD) is one of the most typical forms of dementia and was first proposed by German neuropathologist and psychiatrist Alois Alzheimer in 1906 (Berchtold and Cotman, 1998). AD is a common neurodegenerative disorder that leads to progressive cognitive decline and irreversible memory loss (Association, 2018), eventually causing death from brain failure. It has been reported that 6.2 million people aged 65 years and older in the United States (US) were living with AD in 2021 (2021; Rajan et al., 2021). The number is projected to grow to 82 million in 2030 and 152 million in 2050 (Aminyavari et al., 2019). Additionally, AD deaths have risen markedly according to its death rate, increasing by 145.2% (U.S. Department of Health Human Services, 2020). As a result, health care and long-term care consumption for patients with AD is exceptionally substantial. Alzheimer's and other dementias will cost the nation $355 billion in 2021, while the estimated cost of AD will be more than $1.1 trillion by 2050 in Americans aged 65 and older (2021). Therefore, it has a tremendous impact on the health care system and the quality improvement of the end stage of life.

The main pathological feature of AD is loss of cholinergic neurons, neurofibrillary tangles, senile plaques formed by β-amyloid (Aβ), glial cell activation, and inflammation (Toledo et al., 2015; Weiner et al., 2015; Ossenkoppele et al., 2016; Hampel et al., 2018b; Fu et al., 2019). Thus, the features could be summarized as the cells expressing related proteins expressed by the cells (such as neurons) (Su and Shih, 2015) and pathological properties (such as inflammation, loss of cholinergic neurons, and senile plaques). In particular, the brain's loss of cholinergic neurons and nicotinic acetylcholine receptors (nAChRs) is a significant feature of AD pathology (Yoo et al., 2007). Therefore, the study of the characteristics of cholinergic neurons and cell models could be used to mimic AD pathological damage at the microscopic level. To make effective disease-modifying treatments for AD, sufficient *in vivo* and *in vitro* studies must be conducted to comprehend AD's physiological and pathological mechanisms.

PC12 cells are a rat adrenal pheochromocytoma cell line, a monoclonal cell line transplanted from rat adrenal medulloblastoma by Greene and Tischler in 1976 (Greene and Tischler, 1976). As a catecholamine cells, PC12 cells can synthesize, store and release appropriate amounts of catecholamines (mainly dopamine and norepinephrine). PC12 cells are commonly applied to study neuronal cell death and neurotoxic damage (Greene and Tischler, 1976). Thus, PC12 cells are divided into two types: undifferentiated and differentiated. Undifferentiated PCI2 cells synthesize catecholamines. However, PC12 cells differentiate into sympathetic nerve-like cells under the induction of nerve growth factor (NGF), which are close to neurons in terms of morphology, physiological and biochemical functions, such as growing cell protrusions, forming synapse-like structures, and having electrical excitability properties. Furthermore, under the action of NGF, they can synthesize acetylcholine and form neurite structures (Schubert et al., 1977). Additionally, the PC12 cell membrane has IV-methyl-D-aspartic acid (IV-methyl-D-panic acid, NMDA) receptors (NMDARs, as excitatory amino acid receptors in the central nervous system) that regulate synaptic plasticity, memory, and cognitive ability. The weakened nerve conduction function mediated by NMDARs can lead to brain aging, neuroplasticity damage, and cognitive dysfunction. In addition, NMDARs can interact with amyloid β-peptide/amyloid precursor protein and tau protein (Lin et al., 2014). The experimental evidence of NMDA receptor subunits in PC12 cells is shown in Table 1, and experimental evidence of acetylcholine receptor subtypes expressed in PC12 cells is shown in Table 2. Acetylcholine receptors are divided into cholinergic M and N receptors. Ionotropic nicotinic acetylcholine receptors or metabolizing muscarinic acetylcholine receptors could be activated by acetylcholine in the nervous system. Cholinergic M receptors, also named muscarinic receptors (mAChRs), are called G protein-coupled receptors coupled to G proteins and transduce signals. Furthermore, mAChRs can combine with the endogenous neurotransmitter acetylcholine. Five mAChR subtypes (M1-M5) have been identified, as shown in Table 1 (Nathanson, 1987; Caulfield, 1993; Wess, 1996). Nicotinic receptors greatly impact AD pathophysiological research (Jürgensen and Ferreira, 2010; Hernandez and Dineley, 2012). The degeneration of cholinergic neurons and declining activity of choline-acetyltransferase (ChAT), an enzyme that synthesizes ACh, lead to a decrease in cognition (Davies and Maloney, 1976; Bartus et al., 1982; Ballinger et al., 2016; Shimohama and Kawamata, 2018). Choline acetyltransferase (ChAT) is the main enzyme involved in the biosynthesis of the key neurotransmitter acetylcholine (ACh) from choline and acetyl-CoA (ACoA). Acetylcholine (ACh), as a neurotransmitter, could not only be involved in cognitive functions, such as attention (Howe et al., 2017; Urban-Ciecko et al., 2018), but also participate in plasticity and learning; for example, the release of intermittent choline can adjust the plasticity of different types of synapses in the hippocampus and coordinate pre- and post-synaptic activities (Gu and Yakel, 2011; Gu et al., 2012). Moreover, subunits of nicotinic receptors (as a subtype of cholinergic receptors) are expressed on PC12 cells. These subunits are shown in Table 2, especially α3β4 nAChRs (the salient features of α3β4 nAChRs are the lack of sensitivity of the alkaloid nicotine) (Luetje and Patrick, 1991; Figl et al., 1992; Papke and Heinemann, 1994). In addition, the α3 nAChR, α5 nAChR, α7 nAChR, β2 nAChR and β4 nAChR subunits can be expressed by PC12 cells and combined with other subunits, as shown in Table 2. Thus, PC12 cells have the advantages of being easy to obtain and having a high passage number. Therefore, PC12 cells are widely used to study nerve cell function, differentiation methods, apoptosis and neurotransmitter secretion, and determine potential molecular mechanisms (Spicer and Millhorn, 2003). Meanwhile, PC12 cells are generally used as an ideal cellular model to study pathological molecular mechanisms of AD (Parri et al., 2011).

**Table 1:** Experimental evidence of NMDA receptor subunits existed in PC12 cells.

**Subunit**	**Property**	**Subtype**	**mRNA/ protein**	**Distribution location**	**Penetrating ion**	**Function**	**Subunit combination form**	**Function**	**Receptor relative expression in AD**	**Reference**
NR1	As a functional subunit and an essential subunits of functional NMDA receptors	NR1A-H	+	Prefrontal lobe, hippocampus, cerebellum, brain stem	Ca^2+^, Na^+^	1.Mainly play a role in promoting neuron survival 2. Form functional homomeric channels	NR2,NR3A	Mediate the long-term enhancement of synaptic potency	NR1 subunit transcripts is selectively lacking in pathologically vulnerable areas of AD brain	Foldes et al., 1993; Mcbain and Mayer, 1994; Casado et al., 1996; Laube et al., 1998; Hynd et al., 2004a,b; Peddie et al., 2008; Uhász et al., 2010; Vazhappilly et al., 2010
NR2	Regulatory subunits of NMDA	NR2C	+	Cerebellum	Mg^2+^	Participate in synaptic transmission in specific brain regions	NR1	Mediate the long-term enhancement of synaptic potency		Mcbain and Mayer, 1994; Casado et al., 1996; Chopra, 2004; Uhász et al., 2010; Vazhappilly et al., 2010; Schwartz et al., 2012

The NMDA mainly involved in NR1, NR2 and NR3 three subunits. These subunits could be reacted with other subunits.

**Table 2:** Experimental evidence of acetylcholine receptor subtypes expressed in PC12 cells.

**Acetylcholine receptors subtypes**											
**nAChR subtypes**						**mAChR subtypes**					
**Receptor subunit**	**Function**	**Combination form with other subunits**	**Distribution location**	**Function**	**References**	**Receptor subunit**	**Function**	**Combination form with other subunits**	**Function**	**Distribution location**	**Reference**
α3 nAChR		β4 subunits, β2 subunits	in the adrenal medulla	α3β4 nAChR: Promote the release of catecholamines stimulated by ACh	Boulter et al., 1986, 1990; Rogers et al., 1992; Henderson et al., 1994; Nery et al., 2010; Albillos and Mcintosh, 2018; Criado, 2018	M1 mAChR	Participate in the memory process of the interaction between the cerebral cortex and the hippocampus, and consolidate memory			In the Hippocampus, cortex	Boss et al., 1996; Berkeley and Levey, 2000; Volpato and Holzgrabe, 2018
α5 nAChR	1. not participate in the binding site and is considered an “accessory subunit	α3 and β4 subunits	in the adrenal medulla		Boulter et al., 1990; Rogers et al., 1992; Kuryatov et al., 2008; Nery et al., 2010	M2 mAChR	1. Regulates choline energy nerve pressure and affects nerve circuit function 2. Inhibits neuron excitability, and negative feedback regulates the release of neurotransmitter			In the Pontine-medulla oblongata	Levey et al., 1995; Boss et al., 1996; Mao et al., 2017
α7 nAChR	1. contribute to memory function 2. contribute to synaptic plasticit 3. facilitate neurotransmitter release and dendritic plasticity 4. Activates calcium cation-dependent signaling pathways in cells 5. contributes to the cholinergic anti-inflammatory axis	β2 and β4 subunits	In the hippocampus of the brain area related to memory and cognition	α7β4 nAChR: form a functional heteromeric receptor α7β2 nAChR: causes a significant decrease in agonist-evoked whole-cell current amplitudes	Henderson et al., 1994; Takahashi et al., 1999; Drisdel and Green, 2000; Nery et al., 2010 Wang et al., 2003; Boccia et al., 2010; Parri et al., 2011; Criado et al., 2012; Hernandez and Dineley, 2012; Murray et al., 2012; King et al., 2015, 2018	M3 mAChR	Fear conditioning learning and memory deficits			In the entire central system	Boss et al., 1996; Poulin et al., 2010
β2 nAChR	Participate in the survival of neurons in the brain and the maintenance of cognitive function in aging.	α2,α3,α4, α6 and α7 subunits	In brain	α4β2 nAChR: involved in memory formation and locomotor activity; α7β2 nAChR:form functional receptor	Deneris et al., 1989; Boulter et al., 1990; Rogers et al., 1992; Levin et al., 2002; Nery et al., 2010; Liu et al., 2012; Soll et al., 2013; Moretti et al., 2014	M4 mAChR	Inhibits neuron excitability, and negative feedback regulates the release of neurotransmitter			In the Striatum	Berkeley and Levey, 2000; Wu and Wong, 2006; Mao et al., 2017
β4 nAChR		α3 subunits		α3β4 nAChR: Promote the release of catecholamines stimulated by ACh	Boulter et al., 1990; Rogers et al., 1992; Henderson et al., 1994; Nery et al., 2010; Albillos and Mcintosh, 2018; Criado, 2018	M5 mAChR				In the entire central system	Berkeley and Levey, 2000

The acetylcholine receptor subtypes could be expressed by PC12 cells. The table is divided into two parts, including nicotinic receptor and muscarinic receptor subunits. Among nicotinic receptors, the PC12 cells mainly express α3nAChR, α5nAChR, α7nAChR, β2nAChR, β4nAChR. For muscarinic receptors, muscarinic receptor subunits are expressed in PC12 cells. These subunit receptors could be combined with other subunits.

Therefore, based on the above, PC12 cells have neuronal properties. This study aims to provide a systematic review of the standardization of PC12 cells in AD research. We mainly offer a brief overview of PC12 cells in AD research. The study primarily conducted relevant analyses in the following six aspects: cell source and culture condition, neuronal characteristics induced by differentiation of PC12 cells, the transfection method, the general approach to evaluating the AD cellular model, common damaging agents, and *in vitro* models used in parallel with PC12 cells.

## Main text

In this review, we systemically analyzed the literature covering the use of PC12 cells in AD research. The MeSH Database in PubMed searched keywords, subject headings, subheadings and free words, including “Alzheimer's disease”, “AD”, or “Alzheimer”, and “pheochromocytoma”, “PC12”. 1,003 of 1,717 essays from 1988 Jul 8 to 2021 Feb 10 were included, which were original, available and AD-particular. The first screening was performed according to the following criteria: inclusion of articles with AD specificity and use of PC12 as a cell model (rather than only as a tool to express proteins or genes), and excluding articles not specific for AD, methods papers, reviews, articles with “Alzheimer” as an author, documents that mentioned the cell line from previous studies only in the abstract but did not use it, publications in languages other than English, and not accessible articles (all means are shown in Supplementary material 1).

## The source and culture condition of PC12 cells

It is estimated that PC12 cells are mainly purchased from the American Type Culture Collection (ATCC), institutes, cell banks, universities and donations. Among the pieces of literature mentioned, 50 % of cell sources were not mentioned, 21 % from ATCC, 8 % from institutes, 6 % from cell banks, and 2 % from universities and donations, respectively.

Due to different sources, the culture media composition required for the growth of PC12 cells is different, according to the ratio that appears in the survey, which is listed in Supplementary material 2. RPMI 1640 medium supplemented with 10 % DHS, 5 % BCS, 2 mM L-glutamine, penicillin at 50 units/ml and streptomycin at 50 mg/ml was proposed by ATCC. DMEM supplemented with 10 % FBS, and an institute obtained 0.3% antibiotics; DMEM supplemented with 10 % FBS, 50 units/ml of penicillin and 100 mg/ml of streptomycin was provided by a cell bank. Among systematic studies involving PC12 cells in AD, the basal media is shown in Supplementary material 2. The basal media is essential for the cultivation of PC12 cells, and DMEM (455 out of 820 articles), RPMI 1,640 (267 out of 820 articles) and DMEM/F12 (19 out of 820 articles) are commonly used. In 82 % of articles, basal media was supplemented with supplement serum in Supplementary material 2.

Among the supplement sera, the primarily supplemented sera were FBS, HS and FCS. The changes in FBS ranged from 0.5 to 15 %; among them, the commonly used concentrations of FBS were (280 out of 816 articles) and 5 % (259 out of 816 articles). The content of HS ranged from 0.5 to 20 %, and the commonly used concentrations of HS were 10 % (276 out of 816 articles) and 5 % (151 out of 816 articles). Moreover, the content of FCS ranged from 2.5 to 20 %, and the commonly used concentrations of FCS are 10 % (72 out of 816 articles) and 5 % (58 out of 816 articles). Statistically, in DMEM, the common supplement sera were 10 % FBS, 5 % FBS and 10 % HS. In RPMI 1640 media, the common supplement sera included 10 % HS, 5 % FBS and 10 % FBS. In DMEM/F12 media, the common supplement sera contained 10% FBS, 7% FBS and 10% HS. Among these, 10% FBS, 5% FBS and 10% HS are frequently applied. In addition, the basal media is supplemented with other types of sera. Different concentrations of serum influence the outcome of the experiment (van der Valk et al., 2010), such as cell differentiation (Medina Benavente et al., 2014) and cell transfection. The supplement serum was used either individually or in combination.

Furthermore, the culture media is also involved in other supplements, as shown in Supplementary material 2. According to relevant statistics, antibiotics/antimycotics and L-glutamine are commonly added to basal media. The widely used antibiotics/antimycotics are mixtures of penicillin and streptomycin. The role of antibiotics in the medium is to avoid the production of other bacteria in the culture fluid, affecting the typical living environment of the cultured cells. L-glutamine is a nonessential amino acid (NEAA). L-glutamine and NEAA can participate in cell signaling, gene expression, and metabolic regulation (Deberardinis et al., 2008). The significant difference is that L-glutamine is relatively essential for cells proliferating at high rates (Wu et al., 2014).

Additionally, G418, sodium pyruvate and NEAAs have a crucial influence on cell growth. All plasmids were subcloned into either a pcDNA3 or pcDNA3.1 vector (Invitrogen) containing antibiotic-resistance genes for selection with G418 (Chi et al., 2010). Excess free oxygen and free radicals are eliminated by sodium pyruvate. In addition, the supplements include Glutamax or other components, such as Na_2_CO_3_, NaCl, HEPES, and NaHCO_3_. The effects of additives on cell culture are shown in Supplementary material 2. The types and compositions of culture media are crucial for cell growth and survival, especially disease research, such as on oxidative stress, cell death (Hwang and Lee, 2008; Jäckel et al., 2011) and the metabolic profile (Dietmair et al., 2012). Therefore, it is necessary to systematically acknowledge various metabolic intermediates, ions, serum components and substrates to affect the growth and differentiation of PC12 cells in future disease-association studies.

## The characteristics of PC12 cells after differentiation

PC12 cells are divided into two types: undifferentiated and differentiated. Undifferentiated PC12 cells are small, irregularly shaped, floating cell clusters or scattered lightly attached cells. AD-related studies found that most experiments used undifferentiated PC12 cells because undifferentiated PC12 cells could express nerve growth factor (NGF) receptors and a high transfection capacity (Westerink and Ewing, 2008). Furthermore, PC12 cells contain catecholamine (LDCV) and acetylcholine (LDCV), both of which are found in small transparent follicles (Greene and Tischler, 1976). Moreover, undifferentiated PC12 cells can synthesize acetylcholine and grow neurite structures under the action of NGF (Schubert et al., 1977). In the research using differentiated PC12 cells, differentiated PC12 cells highly express the characteristics of neurons, such as the growth of cell protrusions, the formation of synapse-like structures, and electrical excitability properties. The differentiation condition of PC12 cells for AD research has been described in Table 3 (details are listed in Supplementary material 3). According to statistics, the majority of research used inducers to mediate differentiation. The inducer of differentiation is mainly NGF. NGF plays a vital role in basal forebrain cholinergic neuron differentiation (Thoenen, 1995). NGF is a polypeptide growth factor that has nutritional effects on nerve cells and plays a vital role in nerve cells' growth, differentiation and axon formation (Chao et al., 2006). After adding nerve growth factor (NGF), PC12 cells could be differentiated into sympathetic neurons in the morphology, accompanied by physiological and biochemical changes, and behave like neurons.

**Table 3:** The differentiation condition of PC12 cells for AD research.

**The differentiation condition**				**Number of articles**
**Basal media**	**Supplements**		**Inducer**	
	**Serum**	**Other**		
Not specified	*	*	NGF	92
DMEM	*	*		19
	**√**	*		17
	√	√	NGF	16
	*	*	FGF	1
	*	*	dibutyryl cAMP	1
RPMI1640	√	*	NGF	13
	√	√		9
	*	*		4
	*	√		2
RPMI	√	*	NGF	6
	√	*	FGF	1
	√	*	PMA	1
	√	*	RA	1
Not specified	√	*	NGF	18
	√	√		3
High glucose DMEM	√	*	NGF	1
	*	*		1
DMEM/F12, N2	*	*	NGF	1
N2/DMEM	*	*	NGF	1
DMEM/F-12	√	*	NGF	1

The main inducers included: NGF, FGF, dibutyryl Camp, PMA and RA.

“ ^*^ “ represent not specified; “ √ “ represent specified additives.

NGF, nerve growth factor; RA, retinoic acid; FGF, fibroblast growth factor; PMA, phorbol 12-myristate 13-acetate.

Moreover, PC12 cells treated with or without NGF can synthesize, store, uptake and release catecholamines like sympathetic neurons. However, PC12 cells differentiated by NGF will increase their electrical excitability and neurotransmitter sensitivity (Greene and Tischler, 1976; Greene and Rein, 1977). Moreover, an increasing level of nicotinic cholinergic subtypes or mRNA occurs after differentiation (Henderson et al., 1994). Meanwhile, the ACh-mediated channel activity is also increased. The concentration of NGF is between 50 mg/ml and 100 mg/ml. After culturing PC12 cells in serum-free DMEM medium and adding NGF nerve growth factor, it was found that within a certain period of time, nerve growth factor could promote the differentiation of neurites into neuron-like cells, thereby inhibiting the growth of PC12 cells to a certain extent. The latest research has found that NGF can also promote the proliferation of PCI2 cells, but its effect is quickly overshadowed by the significant differentiation effect of the cells themselves (Mouri and Sako, 2013). When PC12 cells were cultured in an NGF medium (Wiatrak et al., 2020) for 3 days, the cells stopped dividing, grew protrusions and gradually differentiated into cells with characteristics of sympathetic neurons. After 5 days, the protrusions gradually increased and extended to form a network structure, and most of the cells progressively transformed into sympathetic cells. The results showed that after 50 ng/L NGF serum-free culture medium induced by PC12 cells to differentiate for 5 days, the cell diameter increased significantly, the protrusions increased, and the cell differentiation rate reached 72.6%. Furthermore, NGF is a neurotrophic factor that can induce neurite outgrowth in neuronal cells (Alipour et al., 2014). Highly differentiated PC12 cells are directly used in the AD experiment. For example, differentiated rat pheochromocytoma PC12 cells have been cultured in RPMI-1640 medium with 10 % (v/v) fetal bovine serum (FBS), 10 U/ml penicillin, and 10 U/ml streptomycins at 37 °C in 95 % humidified air with 5 % CO_2_ (Ai et al., 2021). Moreover, the highly differentiated PC12 pheochromocytoma cells were cultured in Dulbecco's modified Eagle's medium (DMEM) containing 10 % heat-inactivated fetal bovine serum (FBS), 100 U/ml streptomycin and 100 U/ml penicillin in a humidified 5% CO_2_ and 95% air incubator (Zhao et al., 2018). Moreover, the neurotrophic factors, NGF and FGF, can activate the MEK-ERK and PI3K-AKT pathways, thereby inducing PC12 cell neurite outgrowth (Lai et al., 2011; Wang et al., 2011). In addition, it has been reported that RA of an appropriate concentration can induce the expression of choline acetyltransferase (ChAT) in PC12 cells, thereby forming pseudo cholinergic neurons, which can be used in some experimental studies of AD. Approximately 7–8 days after induction, the cells could form neurites. Moreover, the studies have shown neuronal characteristics upon differentiation media.

## The transfection methods

Differentiation and transfection are two different biological processes of cells. Cell differentiation is a fundamental biological process, and the inducers are essential in addition to the common culture condition. However, distinguished from differentiation, transfection is the transfer of the transfected substance into the cell. Thus, choosing an appropriate cell transfection method is critical to improving the cell transfection rate. The different cell transfection methods used for the AD research are shown in Table 4. According to article statistics, common transfection methods include DNA, RNA, APP and PS. Protein expression was induced by transfection with plasmid DNA as the transfection reagent (Del Toro et al., 2020). Thus, human APP mutation gene-constructed DNA is also applied to transfect PC12 cells. Its main transfection is siRNA transfection in RNA transfection. For cell siRNA transfection, PC12 cells were inoculation in a 6-well plate, and the transfection was performed when the culture was 75–80% confluent. Before transfection, siRNA was incubated with RNAiMAX for 30 min at room temperature. According to the manufacturer's instructions, lipofectamine and reagents (Invitrogen) can transiently transfect PC12 cells containing siRNA (Chen et al., 2019). Furthermore, studies have demonstrated that lipofectamine may be applied as an effective gene carrier for PC12 cells (Lee et al., 2008). Moreover, gene mutations encoded by three essential proteins related to AD may cause familial AD, such as amyloid precursor protein (APP), presenilin 1 (PS1), and presenilin 2 (PS2) (Masters et al., 2015). The APP and PS1 methods are commonly documented by gene transfection. Amyloid (APP) is the amyloid β (Aβ) precursor, a complete membrane protein with a receptor-like structure. In addition, an increase in responsiveness to bFGF stimulation and diminished responsiveness to NGF stimulation could be observed in the transfection of PC12 cells with APP_C100_ gene construct (Sandhu et al., 1996). Studies have shown that antisense PS2 transfection can prevent neuronal growth factor-induced differentiation or apoptosis of amyloid precursor protein expression in PC12 cells during nutritional deficiency (Wolozin et al., 1996). In addition, some studies have shown that transfection with Bcl-2 gene rescued PC12 cells from apoptotic death and oxidative death caused by H_2_O_2_ (Jang and Surh, 2004).

**Table 4:** Different cell transfection methods used for the AD research.

**Transfection methods**	**Number of articles**
DNA	48
RNA	33
APP	21
PS	13
Virus vectors	10
Tau	8
MiR-mimic	8
Lipofectamine	5
GFP	4
Bcl-2	3
PCER	2
Other	37

The table specifies the number of articles that commonly use any transfection method. “Others” refers to articles mentioning other uneasily classified transfection methods.

GFP, Green Fluorescent protein; PCER, phytoceramide.

## The common damaging agents to mimic AD

**Figure 1:**
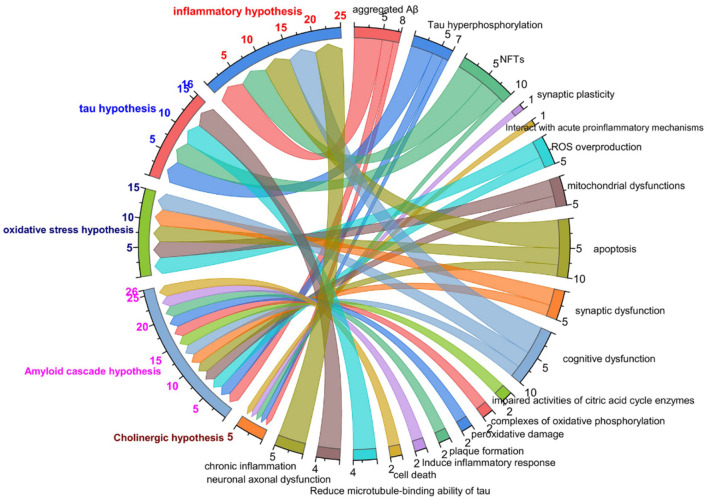
The characteristics of common hypothesis in AD study. Colored text on left side of figure represents cholinergic hypothesis, Aβ hypothesis, Tau protein phosphorylation hypothesis, oxidative stress hypothesis, neuroinflammatory hypothesis, respectively; black text on right side of figure represents relevant pathologic characteristics.

To study the process of disease occurrence, the study of pathological processes is an essential part. The mechanisms of AD are described in the text in Figure 1. In the relevant statistical literature, the “cholinergic hypothesis”, “amyloid hypothesis”, “oxidative stress and free radical damage hypothesis”, “inflammation hypothesis”, and “Tau protein phosphorylation hypothesis” are commonly applied in the pathogenesis of AD. The characteristics of the common hypothesis are shown in Figure 1. The “cholinergic hypothesis” is currently widely accepted (Francis et al., 1999; Hampel et al., 2018a). The loss of cholinergic neuron function is directly related to AD cognitive dysfunction. The amyloid hypothesis was proposed by Hardy and Higgins (1992) and is a widely defined hypothesis in the pathogenesis of AD. According to the amyloid cascade hypothesis, the accumulation of amyloid-β initiates a series of downstream molecular events, driving the pathogenesis of AD (Hardy and Selkoe, 2002). Furthermore, Aβ comprises three forms: a monomer, oligomer and fiber. Abundant evidence has revealed (Bjorklund et al., 2012) that oligomers are the factors that cause cognitive dysfunction in AD. The Aβ oligomer combined with an integrator simultaneously induces the excitement of tyrosine kinase dependence on the N-methyl-D-aspartate (NMDA) receptor. The NMDA receptor is expressed by PC12 cells (shown in Table 1).

**Figure 2:**
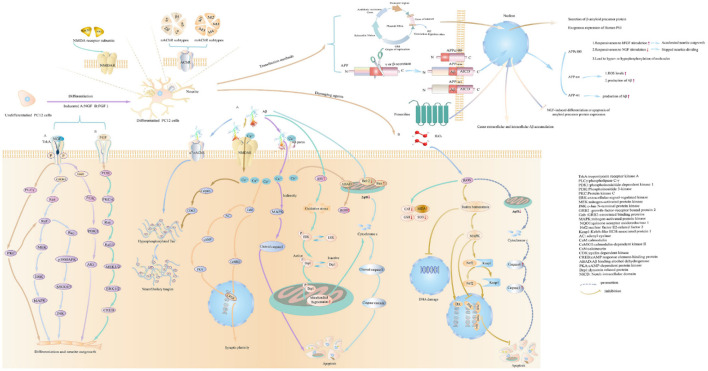
The methods to construct cell models on pathophysiologic processes of AD studies. The figure shows how to cause differentiation, the characteristics of PC12 cells after differentiation, the expression of receptors on PC12 cells, and common damaging agents to mimic AD cell models in AD research.

Understanding the pathological mechanism is essential for AD research, and studies often use drugs to establish PC12 cell injury models. PC12 are considered as sympathetic neuron-like cells, and they are sensitive to apoptosis inducers. Statistics on AD injury cellular models show that the main models are oxidative damage and apoptosis, while inflammation models are relatively rare. The occurrence and development of neurodegenerative diseases are closely related to oxidative stress (Thanan et al., 2014). PC12 rat adrenal pheochromocytoma was used as a cell model, and H_2_O_2_ (free radical trigger) (Li et al., 2018), Aβ and glutamate could be frequently taken as damage agents in AD research. The evaluation indicators and the use of drugs are shown in Figure 2 (The detailed evaluations are shown in Supplementary material 4). Furthermore, the main damaging agents in AD research are shown in Table 5 (The detailed concentration and relevant time of drugs are shown in Supplementary material 5).

**Table 5:** The main damaging agents in AD research.

**Main damaging agents**		**Number of articles**
Aβ peptide	Aβ_25 − 35_	224
	Aβ_1 − 42_	175
	Aβ_1 − 40_	56
	Aβ42	46
	Aβ40	20
Peroxide	H_2_O_2_	92
Amino acid	Glutamate	13
Neurotoxin	6-OHDA	6
Marine toxin	OKA	4
Other		31

The table specifies the number of articles that commonly use any main damaging agents. “Others” refers to articles mentioning other uneasy classified transfection methods. The types of Aβ are frequently Aβ_25 − 35_, Aβ_1 − 42_, Aβ_1 − 40_, Aβ42, and Aβ40; the peroxide is commonly H_2_O_2_; the amino acid is commonly Glutamate; the neurotoxin includes 6-OHDA; the marine toxin includes OKA.

6-OHDA, 6-hydroxydopamine; OKA, okadaic acid; HNE, 4-hydroxynonenal.

Aβ induced injury in PC12 cells is shown in Figure 2. The types of Aβ used mainly include Aβ_25 − 35_, Aβ_1 − 42_ and Aβ_1 − 40_. According to relevant statistics, the most used time of Aβ is 24 h and 48h. The concentrations of Aβ_25 − 35_ is typically 20 μM, 10 μM, 50 μM and 25 μM. Aβ_25 − 35_ induce oxidative damage in PC12 cells, increase intracellular ROS production and reduce mitochondrial membrane potential (Huang et al., 2019). Cell death was classified as apoptosis and necrosis (Núñez, 2011). Apoptosis, the prevalent form of programmed cell death, is essential for maintaining normal cellular homeostasis (Hengartner, 2000), and could be initiated by an extrinsic death receptor pathway or an intrinsic intracellular pathway, each of which is associated with different molecular pathways (Cazanave and Gores, 2009; Ambjørn et al., 2013). Necrosis is regarded as a degenerative phenomenon that lose their membrane integrity after irreversible injury (Weerasekera et al., 2019). Aβ oligomers exert neurotoxicity via induing caspase-3 mediated apoptosis (Kreutzer et al., 2017). Furthermore, the neurotoxicity of Aβ aggregation involves diverse cellular and molecular mechanisms, such as ROS generation, the increase of intracellular Ca^2+^ concentrations, and the induction of apoptosis (Arispe et al., 1993; Behl et al., 1994). The inhibition of neuronal apoptosis might be regarded as one of the effective approaches to preventing Aβ-induced neurotoxicity. The finding demonstrated an increase in apoptosis and necrosis of human umbilical vein endothelial cells with 5 μM Aβ_25 − 35_ treatment for 24 h (Durán-Prado et al., 2014). Additionally, Aβ_25 − 35_ stimulation caused cell apoptosis, and the apoptotic feature is marked by chromatin condensation (Lee et al., 2017). Statistical results show that the common concentrations of Aβ_1 − 42_ are 10, 25, and 20 μM and 50 μM, while the concentrations of Aβ_1 − 40_ are 10, 20, 50, and 25 μM. The studies document that Aβ_1 − 42_ bind with selectivity to nAChR (Li and Buccafusco, 2003). The monomeric and low oligomeric forms of Aβ_1 − 42_ increase the expression of acetylcholinesterase as a consequence of the agonist effect of Aβ_1 − 42_ on the α7 nAChR (Fodero et al., 2004). Meanwhile, human aortic endothelial cells (HAECs) cause toxicity by inducing apoptosis and necrosis after exposure to 10 μM Aβ1-42 for 24 h (Suo et al., 1997). Studies have shown that activating α7 and α4β2 nAChRs reverses the Aβ42-induced hyperexcitability of neurons (Sun et al., 2019). The significant difference is that neurons from Aβ_1 − 40_ toxicity are protected by M1-acetylcholine-muscarinic-receptor (mAChR) activation (Farías et al., 2004). In addition to the common types of Aβ, Aβ42 are commonly used. The relevant statistics indicated that Aβ42 at 10 μM concentrations commonly are used in experiments. The appropriate time for Aβ42 treatment is typically 24 h. Studies confirmed that Aβ42 is the main component of senile plaques in AD (Iwatsubo et al., 1994). Moreover, the Aβ42 oligomer mediates cell oxidative damage (Zheng et al., 2011; Cecarini et al., 2012) and cell apoptosis (Meng et al., 2016). Studies show that the pathological conformation of the tau protein is changed by the Aβ42 monomer (Manassero et al., 2016).

The peroxide is mainly hydrogen peroxide (H_2_O_2_). The use of H_2_O_2_ is at a higher concentration. The commonly used concentrations of H_2_O_2_ are 150 and 100 μM. The appropriate time for H_2_O_2_ is 24 h or 2 h. H_2_O_2_ is frequently used as an inducer to induce cell oxidative stress. Thus, ROS oxidative stress induced by H_2_O_2_ is regarded as an essential factor for causing oxidative cell damage (Gal et al., 2005). It has a wide range of applications in the establishment of apoptosis models. Studies have shown that H_2_O_2_ can induce apoptosis of rat adrenal pheochromocytoma PC12 cells (Lin et al., 2016). Therefore, this study also used H_2_O_2_ as an inducer of PC12 cell apoptosis, which is the general approach used in the PC12 cell model of AD. The mainly-used amino acid is mainly glutamate. The common concentration of glutamate is 100 μM and 10 mM. The relevant time for glutamate is 24 and 10 h. The key to glutamate-induced neurotoxicity is to activate NMDAR and increase Ca^2+^ influx. Furthermore, glutamate could induce the apoptosis model of differentiated PC12 cells (Hu et al., 2016). As a consequence, earlier studies provided evidence that glutamate-induced neurotoxicity is one of the most critical factors leading to the loss of neurons in AD (Bliss and Collingridge, 1993).

Some types are not commonly used. Recently, more evidence showed that a large number of bio-metallics presented in the brains of AD patients (Bush, 2008; Sang et al., 2019; Zhu et al., 2019), such as Cu^2+^, Zn^2+^, Al^3+^ and Fe^3+^. These metal ions can promote the formation of Aβ plaques and NFTs, catalyse the generation of ROS, and cause oxidative damage. Furthermore, these metal ions are present in senile plaques and aggravate the progression of dementia (Ayton et al., 2013; Li et al., 2017). Among these metal ions, zinc and iron can cause tau hyperphosphorylation, and copper ions may be one of the main cationic elements to form senile plaques (Robert et al., 2015). Copper dysregulation is also associated with tau hyperphosphorylation (the main component of NFT) and aggregation. In addition, ferrous and copper ions participate in the Fenton reaction to generate ROS, which aggravates oxidative stress (Barnham and Bush, 2008). One complimentary strategy to study AD in cells is to interfere directly with one of these processes by administering specific compounds with agonistic or antagonistic activity, such as tert-butyl hydroperoxide (oxidative stress), MGO (a potent inducer of AGEs), bafilomycin (inhibitor of vacuolar H^+^ATPase, leading to autophagy dysfunction), thapsigargin (inhibitor of the sarco/endoplasmic reticulum Ca^2+^ATPase, resulting in ER stress and autophagy inhibition), peroxynitrite (a mediator of protein oxidation and nitration, lipid peroxidation, mitochondrial dysfunction, and cell death), and amylin (increased or decreased ERK and Akt phosphorylation in dispersed islets). By simulating the cell model of the disease, the pathological process of the disease can be better understood.

## The general approach to evaluating the AD cell model

In addition to studying the pathogenesis of the disease, it also involves various methods in the pathological process of AD. The common mechanism evaluation indexes of PC12 cells are mainly based on hypotheses in AD research, including the cholinergic hypothesis, oxidative stress hypothesis (including secondary apoptosis), inflammation hypothesis, and Tau protein hyperphosphorylation. A series of different approaches could be widely used to analyse cell apoptosis. Flow cytometry (Dimov et al., 2014) and fluorescence microscopy techniques (Jaber et al., 2020) are broadly applied tools for studying biological processes in cell apoptosis. The fluorescein isothiocyanate (FITC) and propidium iodide (PI) coupled with Annexin V (Annexin V-FITC) could be taken as an approach for detecting the process of apoptosis. Additionally, the Hoechst staining analysis showed that the drug's toxicity to PC12 cells was caused by apoptosis. Among the Hoechst, Hoechst 33,342 has been used to distinguish apoptotic cells from healthy or necrotic cells (Zhivotosky and Orrenius, 2001). Hoechst 33258 has been used to quantitatively determine DNA in biological materials (Saleh et al., 2000). It is essential to determine the corresponding proteins, such as caspase and Bcl-2 proteins (fundamental regulators of apoptosis) (Hayakawa et al., 2016). In particular, caspase activation has been recognized as a critical regulator of the apoptotic pathway (Taylor et al., 2008), which could be associated with the maturation of the pro-inflammatory cytokines IL-1β (Creagh et al., 2003). Therefore, western blotting is usually used to measure protein expression levels (Duan et al., 2019). As a kind of oxygen-containing active substance with high reactivity (Steinbrenner and Sies, 2009), reactive oxygen species (ROS) could induce oxidative stress and cause oxidative damage at excessive levels. Moreover, the generation of ROS can be regarded as the result of the neuroinflammatory cycle, and Aβ peptide is taken as a neuroinflammatory factor to promote ROS generation (Holmes et al., 2011). Therefore, ROS could be considered as one of the indicators of oxidative damage (Mittler, 2017), which affects the generation and accumulation of Aβ (Bonda et al., 2010; Jo et al., 2010; Gwon et al., 2012; He et al., 2017). Moreover, measuring the activity of specific antioxidant enzymes could be applied as a means of assessing oxidative stress, such as superoxide dismutase (SOD), glutathione (GSH) and catalase (CAT). Quantitative real-time PCR (qPCR), immunohistochemistry (ICH) and immunofluorescence (IF) could also be used to measure and evaluate indicators of cellular models. Studies have shown that the miRNA signal in AD can be measured by RT-qPCR (Leidinger et al., 2013). In addition, small non-coding RNA profiles are also analyzed by the RT-qPCR method (Leidinger et al., 2013). However, the physiological and pathological changes in the tissue are often described by immunohistochemistry and immunofluorescence. Immunohistochemistry (ICH) is essential for predicting and detecting minimal residual disease (Loghavi et al., 2015; Kurt et al., 2018). However, immunofluorescence (IF) is a technology for visualizing cell types (Im et al., 2019). Consequently, we could judge if a cellular model establishes successfully through the measurement and analysis of the indicator.

## *In vitro* cellular models used in parallel with PC12 cells

The main characteristic of AD is the loss of neurons (Wang and Zhang, 2020). Therefore, primary cultures of neurons may be considered as reliable models to reveal the underlying molecular mechanisms. However, due to the difficulty of maintaining and introducing experimental variability (Al-Ali et al., 2004) (depending on the age of the source animals or the dissection accuracy), the application of primary cultures of neurons is limited. Although PC12 cells are readily available and simple to culture, there might be some limitations. Undifferentiated PC12 cells were poorly adherent and clustered into a mass (Wiatrak et al., 2020). Importantly, undifferentiated PC12 cells have no neurites and are less responsive to the neurotransmitter of the sympathetic nerve compared with cortical neurons (Wang et al., 2015). Furthermore, undifferentiated PC12 cells with a high passage number are insensitive to damage induced by the cytotoxic agent (Kinarivala et al., 2017). Particularly, PC12 cells above the 16th passage, stimulated by NGF, could exhibit morphological alterations correlated with fibroblast-like phenotype, increase resistance to toxics, accelerate cell division, and lose the differentiation capability (Bothwell et al., 1980; Green et al., 1986; Eveleth and Bradshaw, 1992; Mejía et al., 2013). Meanwhile, undifferentiated PC12 cells are unsuitable for research in neural cells due to low dopamine levels (Wang et al., 2015). However, PC12 cells have the potential of gene mutation, thereby contributing to cause the change of phenotype (Chen et al., 2014). Additionally, PC12 cells originated from pheochromocytoma tumors, so there is no guarantee that the same results will be obtained when studying with *in vivo* models (Grau and Greene, 2012). Owing to the limitations of PC12 cells, so other cell types are also used in many AD studies. In all cited articles, the PC12 cell line was mainly applied in 77% of studies, and 23% of experiments adopted other parallel cell types and PC12 cells. The main alternative cellular models in parallel to PC12 cells in AD research are shown in Table 6 (details are shown in Additional file 6). The parallel cells are primarily sourced from Homo sapiens, Mus musculus, Cricetulus griseus and Cercopithecus aethiops. The Homo sapiens cells mainly comprise SH-SY5Y, HEK293, SK-N-SH and HeLa. The SH-SY5Y (ATCC^®^ CRL-2266™) cells are a subline of the parental line SK-N-SH (ATCC^®^ HTB-11™) (Kovalevich and Langford, 2013). SK-N-SH was subcloned three times: first to SH-SY, then to SH-SY5, and finally to SH-SY5Y. The continuous proliferation, low abundance neuronal markers (Biedler et al., 1978; Påhlman et al., 1984) and the expression of immature neuronal protein are observable characteristics of SH-SY5Y cells. Abundant evidence indicates that SH-SY5Y cells could be differentiated to the cholinergic neuronal phenotype (Kovalevich and Langford, 2013; de Medeiros et al., 2019; Wiedmer et al., 2019). Cholinergic loss is one of the main neuropathological representations of AD (Hampel et al., 2018a). Therefore, the degeneration of cholinergic neurons leads to impaired cognition ability in AD. After differentiation, SH-SY5Y cells could exhibit a phenotype with dopaminergic neurons (Kovalevich and Langford, 2013; Wiedmer et al., 2019) and adrenergic neurons (Kovalevich and Langford, 2013). The loss of dopamine-producing neurons in the substantia nigra pars compacta (Lotharius and Brundin, 2002; Xicoy et al., 2017) is associated with the pathogenesis of PD. During the differentiation process, the increased immunocontent of DJ-1 protein can be found in SH-SY5Y cells. As a neuroprotective protein, DJ-1 could protect dopaminergic neurons from oxidative damage (Björkblom et al., 2013; Choi M. S., et al., 2014; Tanti and Goswami, 2014). Furthermore, the reduction of DJ-1 protein could be extensively related to an early onset of PD (Lopes et al., 2010). Meanwhile, acetylcholine receptors (AChRs) and adrenergic beta2 receptors (B2-ARs) are critical proteins in the neuromuscular junction (NMJ), which is associated with the autophagy and ubiquitin-proteasome system (UPS) in Amyotrophic Lateral Sclerosis (ALS). Thus, SH-SY5Y cells are also broadly applicable to PD and ALS studies (Kovalevich and Langford, 2013; Krishna et al., 2014). Moreover, differentiated SH-SY5Y cells present many neuronal markers at both mRNA and protein levels (Påhlman et al., 1984; Constantinescu et al., 2007). The human embryonic kidney-293(HEK-293) (ATCC^®^ CRL-1573.3) stably expresses rat α3 nAChR and β4 nAChR subunits (Xiao et al., 1998). As a representative cervical carcinoma cell line (Jiang et al., 2010), HeLa cells can express α5 nAChR and β1 nAChR (Calleja-Macias et al., 2009). Furthermore, subcloned M1 mAChR and M2 mAChR genes are permanently expressed in transfected HeLa cells (Pepitoni et al., 1991).

**Table 6:** Use of main alternative cellular models in parallel to PC12 cells in AD research.

**Organism**	**Cell line**	**Tissue**	**Disease**	**Cell type**	**The expression of receptors**													**Number of articles**		**Reference**
					**nAChRs**								**mAChRs**					**Single number**	**Total number**	
					α**3 nAChR**	α**4 nAChR**	α**5 nAChR**	α**6 nAChR**	α**7 nAChR**	β**1 nAChR**	β**2 nAChR**	β**4 nAChR**	**M1 mAChR**	**M2 mAChR**	**M3 mAChR**	**M4 mAChR**	**M5 mAChR**			
Homo sapiens	SH-SY5Y	bone marrow	neuroblastoma						√									71	136	Lukas et al., 1993; Peng et al., 1994
	HEK293	embryonic kidney			√	√			√									35		Kobayashi et al., 2013; Gong et al., 2016
	SK-N-SH		neuroblastoma											√		√		16		Baumgold and White, 1989 Ashkenazi et al., 1989
	HeLa	cervix	adenocarcinoma	epithelial			√			√			√	√				14		Pepitoni et al., 1991; Calleja-Macias et al., 2009
Cricetulus griseus	CHO	ovary											√					14	14	Mullaney et al., 1993
Mus Musculus	Neuro-2a	brain	neuroblastoma	neuroblast		√		√			√							11	11	Xiao et al., 2011; Srinivasan et al., 2012
Rattus norvegicus	C6	brain	glioma	glial cell		√			√		√	√						11	11	Wang and Tang, 2007; Niranjan et al., 2012; Terpinskaya et al., 2021
Cercopithecus aethiops	COS7	kidney		SV40 transformed	√	√					√			√				6	8	Neff et al., 1995; Kobayashi et al., 2013
	COS	kidney		SV40 transformed										√				2		Pals-Rylaarsdam et al., 1997; Vögler et al., 1998

In accordance with organism classification, the table characterizes the use of main alternative cellular models in parallel to PC12 cells. It is divided into five parts. These parts mainly include species, cell line, cell type, and number.

CHO, Naive Chinese hamster ovary; SV40, Simian Virus 40.

The Neuro-2a cell line (ATCC^®^ CCL-131™) is derived from mouse brain neuroblastoma. Neuro-2a cells are utilized in neuronal differentiation, axonal growth and signaling pathways studies (Klebe Rj, 1969; Gómez-Villafuertes et al., 2009; Suzuki et al., 2014). Neuro-2a cells could be differentiated into the cholinergic neuronal phenotype (Gomez et al., 1998) and the dopaminergic neuronal phenotype (Tremblay et al., 2010). Generally, to understand the molecular mechanism of muscarinic function, the CHO cells used to be transfected with the human M1, M2, M3, or M4 mAChR genes (Peralta et al., 1987). Glial cells can be primarily divided into three major classes: microglia, astrocytes, and oligodendrocytes (Moalem and Tracey, 2006). Microglial cells are thought to be the resident macrophages of the central nervous system (CNS) (Dexter and Jenner, 2013). Microgliosis (accumulation of activated microglial) is a characterization of PD (Dexter and Jenner, 2013). The α7 nAChR is ubiquitously expressed in microglial cells (Gotti and Clementi, 2004; Shytle et al., 2004) and astrocytes (Sharma and Vijayaraghavan, 2001; Gotti and Clementi, 2004). Additionally, the expressions of α3, α5 and β4 nAChR have been documented in microglia (Rock et al., 2008). BV-2 is a type of microglial cell derived from C57/BL6 murine. α7 nAChR have been suggested to be the predominant component of immune regulation of the cholinergic anti-inflammatory pathway (Wang et al., 2003, 2004). BV-2 can be activated to release pro-inflammatory cytokines by oxidative stress or inflammatory factors. Such stimulus possibly triggers neurodegenerative disorders such as AD; thus, BV-2 is widely used as an alternative model system for neurodegenerative disease models *in vitro*. In terms of volume and number of cells, astrocytes are the most abundant cells in the CNS (Aldskogius and Kozlova, 1998). C6 cells (ATCC^®^ CCL-10), derived from a rat brain glioma, belong to an astrocyte-like cell line (Shao et al., 2019). At the same time, C6 cells could stimulate acetylcholine synthesis when added to Leibovitz's L-15-CO_2_ neuronal cultures (Patterson and Chun, 1974). These studies showed that α7 nAChRs could be expressed in C6 cells (Wang and Tang, 2007; Niranjan et al., 2012). COS-7 cells (ATCC^®^ CRL-1651™) are transformed with an origin-defective mutant of Simian Virus 40 (SV40) [As a polyomavirus, SV40 has an intricate structure. The capsid is comprised of the major capsid protein VP1 and the minor capsid proteins VP2 and VP3 (Watanabe et al., 2013)]. The α3 nAChRs, α4 nAChRs and β2 nAChRs have been shown to be expressed on the surface of COS-7 cells (Neff et al., 1995). In addition to these common cells, other cells are also adopted in AD research, such as NT2-N, which is derived from the human teratocarcinoma cell line NT2 (ATCC^®^ CRL-1973^TM^). NT2 cells can be irreversibly differentiated to NT2-N cells by retinoic acid (RA) treatment (Pleasure et al., 1992; Neelands et al., 1999). NT2-N cells are characterized by a single axon and multiple dendrites (Novak et al., 1999). Meanwhile, the cholinergic properties may be manifested in the NT2-N cells (Zeller and Strauss, 1995; González-Burguera et al., 2016).

In addition to cellular models, molecular genetic studies have proved that the familial form of AD is closely connected with mutations in the amyloid-β precursor protein (APP), presenilin PSEN1 and PSEN2 (Bilkei-Gorzo, 2014). Moreover, mutations in these three genes are correlated with early-onset AD (Stepanichev, 2020). Thus, the pathophysiological or behavioral characterization of AD could be mimicked by the genetic modification of animals. Genetic modification is usually considered as an ideal approach to mimic AD's pathophysiological or behavioral characterization. The transgenic animal models include single transgenic, such as Tg2576 mice, in which the transgene is the human 695 splice-variant of APP, which contains the double mutation K670 M, N671 L driven by a hamster prion protein gene promoter; double transgenic, such as APPswe/PS1dE9 mice, which carries two transgenes, human APP with the Swedish mutation and human PSEN1 lacking exon 9; and triple-transgenic, such as 3xTg-AD mice, which expresses APP_SWE_, PS1_M146V_, and tau_P301L_ and demonstrates a clear age-dependent onset of AD neuropathology. Undoubtedly, transgenic animal models could play a pivotal role in insights into the pathophysiological basis of AD.

## Discussion

In the past four decades, AD has been the subject of intensive research efforts. In particular, insights into the pathogenesis of AD have been considerably advanced. Patients aged 65 and older waiting for AD-modifying therapy are projected to rose by 34% from 2021 to 2050 (He et al., 2016). One in three seniors dies from Alzheimer's or another dementia (2021). Despite these efforts, no curable or eradicable therapy for AD remains. As of 2020, the drugs for mild to moderate AD or moderate to severe AD treatment that are approved by the FDA mainly include acetylcholinesterase inhibitors, NMDA receptor antagonists, and a fixed-dose combination of an NMDA receptor antagonist and acetylcholinesterase inhibitor, which provide modest and transient cognitive benefits but fail to alter disease process or underlying neurodegeneration (Qaseem et al., 2008; Tan et al., 2014). In the phase I/II/III trial, the continued use of drugs has drawn more attention to passive and active immunotherapy. Compared with the phase II clinical trial (Panza et al., 2019), the drugs numbers in the phase III trial for passive immunotherapy rose by 150%, mainly including aducanumab, solanezumab, gantenerumab and crenezumab. Conversely, the drug numbers in the phase III trial for active immunotherapy fell by 67%. However, aducanumab was the first FDA-approved new drug for the treatment of AD by the “Accelerated Approval pathway” on July 6, 2021. Aducanumab is taken as an anti-amyloid-β antibody, providing potential relief for patients with early AD. As a result, passive immunotherapy offers new insights into future research of new drugs for AD treatment. Therefore, cellular models have served as workhorses in enriching our understanding of the numerous pathophysiological mechanisms associated with disease progression and provide better approaches for mimicking AD in the pre-clinical trial phase of drug development.

The cell culture was markedly affected by the option of media (Weller and Wheeldon, 1982). The cell culture medium plays an essential regulatory role in cell growth and proliferation by providing nutrients and basic materials (Polanco et al., 2020). Meanwhile, the differences in growth indicated that specific cell types require specific media (Bonk et al., 2007). According to the ratio in the survey, DMEM and RPMI 1640 media are frequently used as basal media without the supplemented nutrient (Yuan et al., 2019). The increase in the flattening of the cell and cell-matrix adhesion could be represented after using DMEM culture instead of RPMI 1640 due to the relatively higher concentration of Ca^2+^ in DMEM (Habauzit et al., 2014). When culturing in RPMI 1640, PC12 cells could retain the sensitivity to NGF protein (Greene and Tischler, 1976) and exert minimal neurite extensions (Chua and Lim, 2021). Several supplements involving serum are added to the basal media to obtain the essential growth nutrients (Brown et al., 2019). The serum contains numerous growth factors, lipoproteins and other crucial nutrients for cell growth (Iscove and Melchers, 1978; Hong et al., 2006). The FBS and HS are common serums in the included pieces of literature, and the commonly used concentrations are 10 and 5%.

FBS, as a medium supplement, provides numerous growth factors and nutrients for cells in culture (Schallmoser et al., 2020). When SH-SY5Y cells were cultured in DMEM containing 0, 1 %, 2.5 %, 5 %, and 10 % (v/v) FBS, the result indicated that the number of viable SH-SY5Y cells was notably enhanced with increasing FBS concentration (Ahmadi, 2020). Furthermore, the study has demonstrated that chick cell proliferation rates and cell density increased as serum concentration raised in the range of 5 and 30 % FBS (Ryan, 1979). When human pterygium fibroblasts (as tumor-like transformed cells) were cultured in a medium supplemented with different FBS concentrations (0, 5, 10, and 15 %), the cell confluence reached higher with the increased concentration of FBS (Lopez-Martinez et al., 2019). Thus, we speculated that the serum might provide more essential nutrients and growth factors that facilitate cell survival with increased FBS concentrations. Yet the less proliferation of human pterygium fibroblasts could be found in 15% FBS cultures on day 2 compared to 5 and 10 % FBS cultures, while optimal cell proliferation can be achieved after using 5% FBS on day 3 compared to 10 and 15 % FBS cultures (Lopez-Martinez et al., 2019). Meanwhile, neuronal cells were maintained in the medium containing 1 % FBS, while the proportion of neurons was reduced in the medium containing 10 % FBS (Hashimoto et al., 2000). Additionally, the high concentrations of FBS (20 and 30 %) increased the cellular reprogramming efficacy (RE) of human adipose-derived stem cells (hADSCs) [hADSCs are differentiated into neuron/motoneuron-like cells (Gao et al., 2019)] to generate iPSCs by approximately twice, while the low concentrations of FBS (5%) reduced the RE of hADSCs (Kwon et al., 2016).

HS, containing more immunoglobulins than FBS, has been regarded as a cost-effective alternative to FBS. When equine bronchial fibroblasts cultured in the medium containing FBS, the alpha-smooth muscle actin expression decreased compared with the medium containing FBS (Franke et al., 2014). Although 10% HS was taken as the most common serum, levels between as low as 5 % (Martin and Grishanin, 2003) and as high as 15% (Iuvone et al., 2004) have been applied. Concentrations up to 20 % or even higher were beneficial for cell attachment but suppressed cell proliferation and differentiation, yet concentrations ranging between 5 and 10 % were beneficial for cell proliferation and differentiation (Fedoroff and Hall, 1979). Therefore, speculation could be proposed that the concentrations ranging between 5 and 10% might be appropriate serum concentrations for cell proliferation and differentiation. Selecting a suitable serum concentration and type might be a crucial determinant of the experiments' success. However, antisera production might occur when cells are cultured in the serum-containing medium (Kerbel and Blakeslee, 1976). Additionally, using serum-free culture medium was more beneficial than using serum-containing culture medium for industrial cell culture (Korke et al., 2002). The cell culture medium and the serum are selected according to the characteristics and types of cells.

As immortalized cell lines, PC12 cells derived from a pheochromocytoma of the rat adrenal medulla have been suggested to be an ideal cell for constructing cell models on pathophysiologic processes of AD in *in vitro* studies. The methods to build cell models on pathophysiologic processes of AD studies are shown in Figure 2. PC12 cells could synthesize, store, and release norepinephrine and dopamine (Greene and Tischler, 1976). Statistically, PC12 cells could be divided into undifferentiation and differentiation types. Before differentiation, PC12 cells were poorly adherent and clustered into a mass. Furthermore, the morphological changes of the cell were appreciably altered. Under normal culture conditions, PC12 cells possess morphological, biochemical and physiological characterization of adrenal cells (Wiatrak et al., 2020). After differentiation, PC12 cells are completely adherent and include neuronal phenotypes morphologically and biochemically (Greene and Tischler, 1982). The inductive agent has proven to induce the differentiation of cells. Meanwhile, the NGF receptors could be expressed in PC12 cells. The most frequent and commonly used neurotrophic factor is NGF in a statistical representation of the literature. NGF could present the dual biological role of promoting axon growth and nourishing neurons (Xi et al., 2020). After exposure to NGF, PC12 cells halt proliferation, extend neurites and become electrically excitable (Greene and Tischler, 1976). Furthermore, NGF-induced axon growth in PC12 cells could be mediated by activating PI3K and inactivating GSK-3β (Zhou et al., 2004). Between 4 and 14 days, a noticeable increase in the length of neurites was observed (Wiatrak et al., 2020). After 21 days, the length of neurites reached 500–1,000 μm, and PC12 cells eventually reinitiated proliferation within 3 days after the elimination of NGF (Greene and Tischler, 1976). After differentiation, the activity of the NMDAR1 promoter in PC12 cells could be upregulated by NGF (Bai and Kusiak, 1997). Also, PC12 cells could respond to acetylcholine *via* neuronal-type nicotinic receptors (Casado et al., 1996).

Cell transfection and differentiation are processes that are crucial in developmental biology. Cellular differentiation mainly involves the coordinated regulation of genes by a multitude of cellular pathways, and transfection is the process of artificially introducing nucleic acids (DNA or RNA) into cells. In addition, the main purpose of transfection is to directly investigate the function and products of a gene (Kim and Eberwine, 2010). The general methods are primarily involved in plasmid transfection and gene transfection. Plasmid transfection is frequently accomplished by transfecting DNA or delivering RNA through silencing and overexpressing a wild or mutant gene. DNA transfection provides several advantages compared to RNA transfection, such as targeting specific genomic sites and maintaining transgenes as small episomal plasmids or artificial chromosomes without deleterious consequences (Glover et al., 2005). Plasmid RNA could also be utilized for PC12 cell transfection, in particular for siRNAs, as primers for RNA-dependent RNA polymerase (RdRp), which could synthesize other double-stranded RNA (dsRNA) (Miyagishi and Taira, 2002). The gene transfections in PC12 cells generally include APP and PS. Mutations of PS and APP would result in the autosomal-dominant form in early-onset AD (Lanoiselée et al., 2017; Barthet et al., 2018). In transfected cell lines, mutations in the APP and PS gene could cause extracellular and intracellular Aβ accumulation (Cai et al., 1993; Citron et al., 1997). In particular, the APPsw mutant in PC12 cells could induce oxidative stress and eventually lead to apoptotic cells by activating c-Jun N-terminal kinase and caspase 3 and reducing caspase 9 activity (Marques et al., 2003). In addition, the gene Presenilin mutations in PC12 cells could increase apoptotic cells induced by Aβ or trophic factor withdrawal, especially PS1 (Guo et al., 1997) and PS2 (Wolozin et al., 1996).

Cell differentiation or transfection is also recognized as a fundamental means of establishing a successful cell model. The establishment of cellular models is also critically needed to mimic the pathophysiological hallmark of AD. Among the popularly accepted pathology of AD, the cholinergic system plays an essential role in neuronal function, such as memory, learning, and plasticity. In particular, the loss of acetylcholine in the hippocampus and neocortex is closely associated with AD (Bartus et al., 1982; Babic, 1999). Moreover, cholinergic lesions manifest complex interactions with pathological hallmarks of AD, such as amyloid-β plaques, neurofibrillary tangles and neuroinflammation (Hampel et al., 2018a). Thus, Aβ is regarded as a common damaging agent because it can cause oxidative stress, mitochondrial dysfunction, and apoptosis (Leuner et al., 2012). The Aβ oligomers that precede plaque formation have drawn more attention beyond Aβ fibrils of the insoluble plaques in the research of Aβ toxicity (Bjorklund et al., 2012). Increasing evidence has implicated that Aβ oligomers are viewed as the primary agents for tau hyperphosphorylation (De Felice et al., 2008; Jin et al., 2011), synaptic dysfunction (Benilova et al., 2012) and cellular toxicity (Haass and Selkoe, 2007; Glabe, 2008). Our study provides evidence that Aβ_25 − 35_, Aβ_1 − 42_ and Aβ_1 − 40_ are generally considered as the main damage agents in the amyloid cascade hypothesis. The amino acid sequence and identified methods regarding Aβ peptide are shown in Table 7. According to the literature, the damage action time of 24 h has been generally adopted, while the concentrations of 20, 10, and 25 μM of Aβ_25 − 35_, Aβ_1 − 42_ and Aβ_1 − 40_ have been applied frequently, respectively. As an essential form of ROS (Matsushita et al., 2020), H_2_O_2_ is widely used as the inducer for oxidative damage and apoptotic cell death (Maroto and Perez-Polo, 1997). Some intracellular apoptotic pathways, such as the PI3K/Akt signal pathway (Li et al., 2020), could be activated by excessive ROS. The application of H_2_O_2_ in PC12 cells could be considered as a proper model system for analyzing antioxidants and the apoptosis prevention mechanism (Hu et al., 2014; Chen et al., 2015), especially regulating apoptosis-related proteins, such as anti-apoptosis/pro-apoptosis proteins, and caspases (Jin et al., 2013). The oxidative damage action time of 24 h and the concentrations of 100 and 150 μM are typically adopted. Besides, H_2_O_2_ could increase γ-secretase activity to facilitate Aβ production through c-Jun N-terminal kinase (Shen et al., 2008). In addition, evidence suggests that neuropathological hallmarks of AD include stress-induced hyperphosphorylation of tau (Krstic and Knuesel, 2013). The hyperphosphorylated tau could make a contribution to synaptic dysfunction, mitochondrial dysfunction and cognitive impairment (Di et al., 2016). OKA acts as a potent and highly selective PP2A inhibitor to induce hyperphosphorylated tau (Kamat et al., 2014). The damage action time of 4 h and concentrations of 90 nM are frequently used within the literature. Based on the associated hypothesis of AD, the damaging agent-induced cellular models provide an excellent approach to study the pathological hallmarks of AD.

**Table 7:** The amino acid sequence and identified methods for Aβ peptide.

**Types**	**Molecularformula**	**Molecular**	**Amino acid sequence**	**3D Structure**	**Structure-neurotoxicity**					**Characteristic**						**Identified methods**									**Reference**
					**I31-V40**	**CHC**	**I31-V40 C**	**alpha-helixconformation**	β**-sheet conformation**	**active region of A**β	**proteolytic cleavage**	**CHC**	**G37-G38**	**C-terminal amino acidsIle41-Ala42**	β**-strand structure involving Ala-2–Phe-4**	**CD**	**NMR**	**AFM**	**ThT**	**EFM**	**SKM**	**cryo-EM**	**IEM**	**Other**	
Aβ_25 − 35_	C45H81N13O14S	1060.27	H-Gly25-Ser-Asn-Lys-Gly-Ala-Ile-Ile-Gly-Leu-Met35-OH	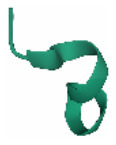 PDB (1QWP)					√	√	√					√	√	√	√	√	√				Yankner et al., 1990; Terzi et al., 1994; Sato et al., 1995; Kubo et al., 2002; Naldi et al., 2012; Chen et al., 2017
Aβ_1 − 42_	C203H311N55O60S	4514.1	H-Asp-Ala-Glu-Phe-Arg-His-Asp-Ser-Gly-Tyr-Glu-Val-His-His-Gln-Lys-Leu-Val-Phe-Phe-Ala-Glu-Asp-Val-Gly-Ser-Asn-Lys-Gly-Ala-Ile-Ile-Gly-Leu-Met-Val-Gly-Gly-Val-Val-Ile-Ala-OH	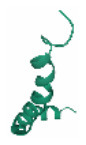 PDB (1IYT)	√	√	√		√			√	√	√		√	√					√	√	colorimetric, fluorometric methods, differential interference contrast optics, laser scanning confocal immunofluorescence	Naiki and Gejyo, 1999; Anderson et al., 2004; Urbanc et al., 2004, 2010; Inouye and Kirschner, 2005; Bartolini et al., 2007; Middleton, 2007; Ahmed et al., 2010; Chen et al., 2017
Aβ_1 − 40_	C194H295N53O58S1	**4329.9**	**H-Asp-Ala-Glu-Phe-Gly-His-Asp-Ser-Gly-Phe-Glu-Val-Arg-His-Gln-Lys-Leu-Val-Phe-Phe-Ala-Glu-Asp-Val-Gly-Ser-Asn-Lys-Gly-Ala-Ile-Ile-Gly-Leu-Met-Val-Gly-Gly-Val-Val-OH**	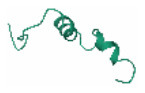 PDB (1AML)	√	√	√	√							√		√		√			√			Urbanc et al., 2004, 2010; Williams et al., 2005; Sachse et al., 2006; Meinhardt et al., 2009; Bertini et al., 2011; Naldi et al., 2012; Chen et al., 2017

The table mainly describes the molecular and molecular formula, amino acid sequence, structure and identified methods in Aβ peptide. To present the results in a systematic manner, the peptide is segmented as follows: (i) Asp-1–Lys-16 is the N-terminal region; (ii) Leu-17–Ala-21 is the central hydrophobic cluster (CHC); (iii) Glu-22–Gly-29 is the turn A (TRA) region; (iv) Ala-30–Met-35 is the mid-hydrophobic region (MHR); (v) Val-36–Val-39 is the turn B (TRB) region; and (vi) Val-40 or Val-40–Ala-42 is the C-terminal region (CTR).

EFM, electrostatic force microscopy; AFM, atomic force microscopy; SKM, scanning Kelvin microscopy; cryo-EM, cryo-electron.

In an attempt to better reflect the AD-related pathological features, selecting appropriate verifiable methods and indicators for the analysis is equally crucial to establishing successful cellular models. Levels of anti-apoptotic Bcl-2, pro-apoptotic Bax and caspases have been shown to correlate with apoptosis in PC12 cells *in vitro*. Studies show that PC12 cell apoptosis could be prevented by downregulating the caspase-3 protein and inhibiting the Bax/Bcl-2 ratio (Hao et al., 2015). The ROS, MDA and antioxidant enzyme activities are closely correlated with oxidative stress. DCFH-DA essay is regarded as the dominant method for determining the level of ROS in PC12 cells (Eruslanov and Kusmartsev, 2010), and the increase in ROS leads to damaged mitochondria and activation of the apoptotic cascade (Zeng et al., 2017). Antioxidant enzymes, such as SOD, CAT, and GPx, are suggested to be the oxidative defense system in PC12 cells (Poprac et al., 2017). ACh has a crucial role in the pathophysiology of AD. The level of ACh in the brain is regulated by AChE and butyrylcholinesterase (BChE). The decreased expression of pro-inflammatory cytokines, such as interleukin-1-beta converting enzyme (IL-1β) and tumor necrosis factor-alpha (TNF-α), are taken as common indicators of inflammation (Ma et al., 2019).

However, not all aspects of AD pathogenesis can be covered in PC12 cells. As a result, the *in vitro* models used in parallel with PC12 cells also play an essential role in AD studies. Of the articles analyzed, the PC12 cells are mainly involved in 77% of the literature. The other 23% of experiments adopt other parallel cell types and PC12 cells, including (mainly cortical) primary neurons, the SH-SY5Y cell line, HEK293, and Neuro-2a cell lines. The expression of nAChR or mAChR could be found on the surface of these cells. The loss of cholinergic neurons is tightly associated with AD pathology. These cells would provide a global overview of the landscape and diversity of cellular models that can be used in AD. These cellular models also presented their own limitations. The primary neuron possessed a limited life span (typically days to weeks) (Li, 2011) and needed to be generated from the embryonic or early postnatal brain (Sahu et al., 2019). The differentiation state of SH-SY5Y cells was undefined and ranged from tumor tissue state (neuroblastoma) to neural progenitor cells or post-mitotic neurons (Feles et al., 2022). Although differentiated SH-SY5Y cells were commonly used to study the growth of neuronal processes (Kovalevich and Langford, 2013; Shipley et al., 2016; Peng et al., 2021), mechanisms involving in neuronal function and excitability cannot be easily compared with the physiological state derived from brain tissue-derived cells. Due to deficient DNA mismatch repair, HEK293 cells were especially vulnerable to genotype drift caused by external disruptions (Panigrahi et al., 2012). However, compared with cerebellar granule neurons, voltage-gated sodium channel expression in Neuro-2a cells was approximately 20-fold lower, and NMDAR expression was also not expressed in Neuro-2a cells (Lepage et al., 2005). Meanwhile, an increase in dopamine neuronal characteristics was manifested only after dbcAMP induced differentiation of Neuro-2a cells (Tremblay et al., 2010). In addition, the cellular metabolism of CHO cells was altered during long-term culture (LTC), including extracellular alanine accumulation and enhanced utilization of glucose and lactate (Bailey et al., 2012). Furthermore, CHO cells was also involved a slower growth rate and low-stress resistance (Fischer et al., 2015). For microglia cells, the limitations probably contributed to the cognitive loss and neuronal damage (Giulian, 1999). BV-2 cells have weaker responses to LPS and interferon-gamma stimuli compared with primary mouse microglia (Stohwasser et al., 2000). Due to the complexity and diversity of AD pathogenesis, these cell models only partly imitate AD pathological microscopic characterizations.

In addition to the above cells, induced pluripotent stem cells (iPSCs), miRNA-induced neurons, neuronal cells, Brain microvascular endothelial cells (BMECs), and genetically engineered cells are broadly applied to construct the AD model. The iPSCs are currently utilized for establishing familial AD cellular models *in vitro* (Amin et al., 2019). The iPSC from AD patients carried the same AD-causing gene and obtained the same phenotype and function as AD neuronal cells. The iPSC, derived from mouse embryonic fibroblasts, could be generated by transduction of transcription factors with Oct3 /4, Sox2, c-Myc, and Klf4 (Takahashi et al., 2007). Fibroblast of iPSC obtained from AD patients can be used for orthotopic modeling, whose procedure involves seeding tumor cell lines or patient-derived cell xenografts into animal models (Brodaczewska et al., 2016). However, *in vitro* models of iPSC lack cellular diversity and possess structural complexity in two-dimensional model (Majolo et al., 2019). The microRNA (miRNA)-induced neurons have great potential as neurotoxicity screening. The miRNA plays an essential role in neuronal development, proliferation and differentiation, apoptosis, and homeostasis (Kaur et al., 2012; Denk et al., 2015; Sun and Shi, 2015). The hippocampal neuronal cells and cerebral cortical neural cells are considered as reliable AD cellular models to unravel pathological changes of AD. Hippocampus degeneration is closely associated with learning and memory dysfunction in AD patients (Fujishiro et al., 2006). The cerebral cortex of AD patients appear abundant neurofibrillary lesions and neuritic plaques (Fitzpatrick et al., 2017). Yet the application of neuronal cells was precluded owing to the inaccessibility and lack of proliferation. BMECs are also used to establish the AD cellular model for the following two reasons. BMECs are taken as a crucial component of blood brain barrier (BBB) (Zlokovic, 2008). BBB decomposition is thought to be an initiating factor in the pathogenesis of AD (Yoon et al., 2021). Furthermore, APP and β-secretase enzyme could also be found in BMECs (Bourassa et al., 2019). APP and β-secretase enzyme are co-localized to the site of intracellular Aβ production (Long and Holtzman, 2019). With the development of biomedical and genetic engineering technologies, genetically engineered cells are used to establish the AD cellular model. AD genetically engineered cell lines, particularly PC12, SK-N-SH, and A172, were constructed by the cloned amyloid cDNA that contains a region encoding A4 (beta-polypeptide) amino acids along with recently developed tumor virus vectors derived from simian virus 40 (Marotta et al., 1989). The stable Myh9 in PC12 cells were knocked out by CRISPR/Cas9 nucleases (Wang et al., 2017, 2020), powerful genome engineering tools (Rose et al., 2020). The CRISPR/Cas9-mediated genome editing technology has become a promising approach for the choice of gene targeting (Pelletier et al., 2015), which is broadly applied for gene editing in multiple cell types and organisms (Platt et al., 2014).

Additionally, these cell model systems are also used to mimic AD pathophysiology, discover biomarkers or potent therapeutic drugs, or conduct high throughput drug screening. Traditionally, 2D cell culture models do not cover the complex cellular microenvironments *in vivo*; thus, they are insufficient to predict *in vivo* efficacy and toxicity. More advanced 3D cell culture models (e.g., organoids, microtissues, spheroids) have been adopted to mimic not only the microenvironment (Lee et al., 2022) but also gene expression and functional characteristics of tissues *in vivo* (Edmondson et al., 2014). 3D-culture emulate complex cell interactions, multicellular architecture, cell-cell interactions and physical microenvironment of interactions (Ingber, 2018). The 3D cell culture models of human cell lines and primary cells provide a promising approach to improve drug development and screening. In *in vitro* 3D culture system, neuronal cells differentiated by the iPSCs obtained from familial AD patients accelerate Aβ plaques and neurofibrillary tangles associated (Choi S. H., et al., 2014). As is mentioned above, the 3D cell culture model might provide a promising insight to mimic the complex pathogenesis of AD.

In addition to cellular models, animal models could be widely adapted in the earlier preclinic stage of drug development. Particularly, transgenic animal models contain linear human chromosomes and a similar number of genes as humans (Waterston et al., 2002). In order to dissect the mechanisms underlying AD, transgenic animal models present an efficient pathophysiological process and early typical symptoms in AD, such as memory impairments, Aβ pathology and neuroinflammation (Bilkei-Gorzo, 2014; Saito et al., 2014). At the same time, transgenic models could also be thought as the identification and validation of drug targets for AD (Zahs and Ashe, 2010).

Additionally, metabolomics is widely applied in AD pathogenesis (Arnold et al., 2020). The levels of metabolites were significantly different among the brain tissue and liver of AD wild-type and transgenic mice (Wang et al., 2019). The unusual metabolic pathway of amino acid metabolism, energy metabolism, and gut microbiota could be found in the AD rat model through the metabolomics. Moreover, features of AD could be reproduced by neuronal reprogramming of fibroblasts from familial AD patients into functional neurons (Qiang et al., 2011). Cell-direct reprogramming could convert one fully differentiated cells into another differentiated state (Riva et al., 2022), such as directly reprogramming fibroblasts into neural stem cells (Han et al., 2016), neuronal cells (Treutlein et al., 2016), or mature cardiomyocytes (Herrero and Bernad, 2016). Cell-direct reprogramming technology could supplement iPSC technology and enhance the differentiation of iPSCs via directly reprogramming somatic cells into iPSCs and lineage-restricted stem cells (Ring et al., 2012). Therefore, due to a wide range of sources and a high conversion rate on somatic cells, direct reprogramming technology of somatic cells could outperform traditional iPSC technology. Based on above all, it is speculated that somatic cell reprogramming may have tremendous potential for disease modeling.

## Conclusion

Due to the multifactorial and complex pathogenesis of AD, the biomarkers associated with the AD pathogenic mechanism may be crucial for the early detection and prevention of AD (Kapogiannis et al., 2019). Decreased Aβ_1 − 42_ (as a marker of amyloids) and increased tau (as a marker of neuronal injury) levels in cerebrospinal fluid (CSF) are the most widely used characteristic biomarkers of AD patients (Sunderland et al., 2003; van Maurik et al., 2017), and low CSF levels of Aβ_1 − 42_ are associated with intracranial amyloid deposition, while high CSF levels of phospho-tau (p-tau) are correlated with tau-associated neurofibrillary tangles (Hampel et al., 2018c).

Research on biomarkers associated with AD *in vitro* cell models would provide valuable insights into the therapeutic intervention of AD. The cell models can mimic multi-pathophysiological features underlying AD pathogenesis in the micro-changes and processes, which can also be regarded as a promising means for potential therapeutic drug screening. PC12 cells are utilized extensively as a neuronal model in neuroscience research. PC12 cells could synthesize, store, and release norepinephrine and dopamine compared to other cell models. Furthermore, neurotransmitter receptors associated with AD are present on the surface of the PC12 cell membrane (Westerink and Ewing, 2008), especially NMDARs and cholinergic receptors. The attenuation of neurotransmission mediated by NMDAR can lead to neuroplasticity damage and cognitive dysfunction in the aging brain (Lin et al., 2014). NMDAR activation could inhibit Aβ production and release by stimulating nonamyloidogenic APP processes (Marcello et al., 2007; Hoey et al., 2009). The expression of NR1 and NR2 subunits could be manifested in PC12 cells. Meanwhile, the nicotinic receptor-mediated neurotransmitter ACh is closely associated with AD pathology. The degeneration of cholinergic neurons and declining activity of choline-acetyltransferase (ChAT) have contributions to a decrease in cognition (Davies and Maloney, 1976; Bartus et al., 1982; Ballinger et al., 2016; Shimohama and Kawamata, 2018). The α3 nAChR, α5 nAChR, α7 nAChR, β2 nAChR and β4 nAChR subunits can be expressed by PC12 cells. In addition, PC12 cells differentiate into sympathetic nerve-like cells under the induction of NGF and could be sub-cultured indefinitely. Therefore, PC12 cells are not only systematically used to study nerve cell function, differentiation, apoptosis and neurotransmitter secretion (Spicer and Millhorn, 2003), but also usually as an ideal cellular model to extensively explore the pathological molecular mechanisms of AD (Parri et al., 2011).

The development of cellular models reflecting the microscopic pathophysiological features of AD is likely to offer new insights, which also may benefit for exploring the potential therapeutic targets.

## Data availability statement

The original contributions presented in the study are included in the article/Supplementary material, further inquiries can be directed to the corresponding authors.

## Author contributions

DX, TD, ZZ, YX, and TS contributed to the conception and design of this study, manuscript preparation, and reviewed the final version. DX contributed to the data extraction and analysis. All authors read and approved the final manuscript.
